# Which features best differentiate Sjögren’s-related from non-Sjögren’s-related dry eye disease?

**DOI:** 10.1007/s00417-026-07334-w

**Published:** 2026-07-08

**Authors:** Germán Mejía-Salgado, William Rojas-Carabali, Carlos Cifuentes-González, Juanita Téllez-Zambrano, Blanca Aguilar-Barrera, Laura Daniela Rodriguez-Camelo, Santiago Espinosa-Lugo, Guillermo Marroquín-Gómez, Martha Lucía Moreno-Pardo, Juliana Tirado-Ángel, Anat Galor, Alejandra de-la-Torre

**Affiliations:** 1 Neuroscience Research Group (NEUROS), Neurovitae Center for Neuroscience, Institute of Translational Medicine (IMT), Escuela de Medicina y Ciencias de la Salud, Universidad del Rosario, Carrera 24 # 63 C 69, Bogotá, Colombia; 2 Clinical Research Group; Health Sciences Faculty, Universidad Autónoma de Bucaramanga (UNAB), Bucaramanga, Colombia; 3 Colombian Visual Science and Translational Eye Research Institute (CERI), Centre of Excellence in eye inflammation, Bogotá, Colombia; 4 Ophthalmology Interest Group Universidad del Rosario. (OIG UR). Escuela de Medicina y Ciencias de la Salud, Universidad del Rosario, Bogotá, Colombia; 5 HORUS, Grupo Oftalmológico. Cra 19b #84-65, Bogotá, Colombia; 6 Bascom Palmer Eye Institute, University of Miami, Surgical Services, Miami Veterans Administration, Miami, FL USA

## Abstract

**Purpose:**

To identify which features best differentiate Sjögren’s-related from non-Sjögren’s related dry eye disease (DED) using machine-learning models.

**Methods:**

A retrospective study was conducted on 324 individuals with DED based on Dry Eye Workshop-II criteria, including 162 with and 162 without Sjögren’s disease (SjD). One eye per subject was randomly selected. Demographics, Ocular Surface Disease Index (OSDI), and clinical signs were collected. A symptom–sign discordance score was calculated (range − 1 to + 1), positive values indicated greater symptom burden relative to signs, and negative values indicated greater clinical signs relative to symptoms. Multiple machine learning models were developed, and the one with the highest accuracy was selected as optimal. Feature importance, Weight of Evidence (WoE), and Information Value (IV) were computed for the top five predictors in the optimal model.

**Results:**

The mean age was 47.4 years, and 82% of individuals were female. Compared to the non-SjD group, those with SjD had lower OSDI scores (31.8 vs. 44.6, *p* < 0.001) despite higher ocular surface staining (OSS) (Oxford IV 5.5% vs. 4.9, *p* = 0.015), shorter non-invasive tear break-up time (NIBUT) (8.6 vs. 10.6 s), lower Schirmer values (7.5 vs. 14.4 mm), and a higher rate of positive InflammaDry tests (73.4% vs. 25.3%, *p* < 0.001). Discordance scores for OSS (− 0.34 vs. +0.09, *p* < 0.001), NIBUT (− 0.60 vs. −0.39, *p* < 0.001), and Schirmer (− 0.46 vs. −0.39, *p* = 0.003) were more negative in the SjD compared to the non-SjD group, indicating that objective signs exceeded symptoms across all three metrics in the SjD group. The optimal model (accuracy = 0.96) identified the most predictive features of SjD-related DED as a positive InflammaDry test (WoE: 1.034, IV: 0.45), OSS discordance (score < − 0.5) (WoE: 1.376, IV: 0.42), NIBUT discordance (score < − 0.5) (WoE: 2.079, IV: 0.22), Schirmer test < 5 mm/5 min (WoE: 1.007, IV: 0.18), and Schirmer discordance (score < − 0.5) (WoE: 0.746, IV: 0.09).

**Conclusion:**

SjD-related DED is characterized by ocular surface inflammation, symptom-sign discordance with signs outweighing symptoms, and a Schirmer test < 5 mm/5 min.

**Supplementary Information:**

The online version contains supplementary material available at https://doi.org/10.1007/s00417-026-07334-w.

## Key message

*What is known*

Sjogren’s disease is a major cause of dry eye disease (DED) and has a heterogeneous clinical presentation.

*What is new*

Using machine learning, this study identified a positive InflammaDry test, a pattern of symptom–sign discordance characterized by greater objective disease severity (worse signs) relative to subject-reported symptoms, and a Schirmer test <5 mm/5 min as the strongest predictors of Sjögren’s-related DED.

These findings support the use of non-invasive clinical data to differentiate Sjögren’s-related from non-Sjögren’s-related DED with high accuracy.

**Supplementary Information:**

The online version contains supplementary material available at https://doi.org/10.1007/s00417-026-07334-w.

## Introduction

Sjögren’s disease (SjD) is a chronic autoimmune disorder characterized by lymphocytic infiltration of exocrine glands, leading to hallmark symptoms such as dry eyes and dry mouth [1]. It can occur as a primary condition or in association with other autoimmune diseases, including rheumatoid arthritis and systemic lupus erythematosus [1]. SjD is the second most prevalent autoimmune rheumatic disorder after rheumatoid arthritis, with an estimated global prevalence of 0.06% [2]. Importantly, SjD is associated with substantial morbidity. Beyond dry eyes, ocular complications may include severe manifestations such as corneal melt, cicatrizing conjunctivitis, and scleritis, while systemic involvement can affect multiple organs, including the nervous system, kidneys, and lungs [3].

Despite its clinical relevance, SjD remains underdiagnosed or diagnosed late, with up to 75% of individuals experiencing a significant delay before diagnosis, with a mean time to diagnosis of ~ 3.5 years [4, 5]. This is largely attributable to limitations in current classification criteria, which depend on invasive procedures such as labial salivary gland biopsy or on the detection of specific serologic markers (anti-Ro/SSA antibodies) [6]. These diagnostic tools present important challenges; for example, biopsies are not always feasible in routine clinical practice, and serologic markers may be absent in a substantial proportion of individuals with clinical features suggestive of SjD [6]. As a result, there is a critical need for alternative, accessible, and non-invasive approaches to improve SjD identification.

Dry eye disease (DED) is one of the most common manifestations of SjD, and its findings may enable earlier identification, as the clinical tests used to evaluate DED are widely available. Previous studies have shown that SjD-related DED exhibits a distinct clinical phenotype, including greater ocular surface damage and a notable discordance between symptoms and signs, with signs outweighing symptoms [7, 8]. In parallel, advances in artificial intelligence (AI) have demonstrated that clinically accessible data—particularly ocular features—can be leveraged to identify systemic diseases with high accuracy. For example, a large-scale study involving 145,832 subjects demonstrated that deep learning models based on external eye photographs significantly outperformed models relying on clinicodemographic variables (e.g., age, sex, race/ethnicity, and diabetes duration) in detecting diabetic retinopathy, diabetic macular edema, and poor glycemic control [9]. These findings highlight the potential of ocular data as a non-invasive surrogate for detecting systemic diseases.

In this context, ocular surface parameters may serve as valuable, non-invasive markers for detecting SjD. Therefore, this study aimed to identify the ocular symptoms and signs that best differentiate SjD-related from non-SjD-related DED using machine learning models.

## Methods

### Setting and clinical protocol

This study included individuals evaluated at a specialized dry eye diagnostic center in Bogotá, Colombia. Subjects assessed at this center were referred from both private and public ophthalmology practices for diagnostic confirmation of DED and detailed characterization of ocular surface findings. The center provides a comprehensive, standardized diagnostic evaluation of the ocular surface and issues a final diagnostic assessment (yes or no). If yes, subjects were categorized into aqueous-deficient DED, evaporative DED, or mixed-component DED, and then referred back to their primary ophthalmologist. Importantly, this center functions exclusively as a diagnostic referral unit. No therapeutic decisions are made, and no treatments are initiated, modified, or followed at this facility. All treatment decisions and longitudinal follow-up are conducted by the referring ophthalmologist outside the clinic.

As part of the clinical protocol, all individuals first underwent a standard medical evaluation focusing on prior diagnoses of systemic diseases. The conditions captured included: SjD, connective tissue diseases, diabetes mellitus, rosacea, thyroid diseases, psychiatric disorders, atopic diseases, and migraine. Additionally, a comprehensive ophthalmological history was obtained, including prior refractive, cataract, or eyelid surgery and contact lens use. Importantly, systemic and ocular conditions were recorded from individual-reported medical history as documented in the clinical record; no additional diagnostic testing was performed at the center to confirm the diagnoses.

Also, as part of the clinical protocol, all participants completed the Ocular Surface Disease Index (OSDI) questionnaire and underwent all ocular surface tests in the following order: blink frequency, tear film osmolarity, non-invasive tear break-up time (NIBUT), lipid layer thickness, tear meniscus height (TMH), ocular surface staining (OSS) using the Oxford grading scheme [10], bulbar nasal and temporal redness based on Oculus [11], Meibomian gland (MG) expressibility, based on Shimazaki et al. [12], and the degree of MG dropout, which was graded according to the Pult retrograde scale [13], MMP-9 levels, and Schirmer’s test with anesthesia. Evaluations were performed using the Topcon DS3 anterior segment camera, the TearLab Osmolarity System, the Oculus Keratograph 5 M, the InflammaDry test, and traditional Schirmer’s strips by two trained optometrists. Detailed descriptions of the equipment and measurement procedures for each variable are provided in Supplementary Material 1.

### Dry eye disease diagnosis

For the current study, clinical data were extracted by a data scientist, and individuals were retrospectively classified as having DED (yes/no) based on DEWS II criteria, defined by the presence of symptoms (OSDI ≥ 13) and at least one sign of disrupted homeostasis (NIBUT < 10 s, osmolarity *≥* 308 mOsm/L, OSS with more than five corneal spots, more than nine conjunctival spots, or lid margin involvement greater than two mm in length and 25% in width) [14]. Those with missing data in the variables necessary for diagnosing DED (OSDI, NIBUT osmolarity, OSS, and lid margin involvement) were excluded (*n* = 123).

### Selection of subjects for inclusion in the current study

From a total of 2,350 eyes from 2,350 individuals, 2,050 were retrospectively diagnosed with DED. Among these, individuals with SjD were identified by the same data scientist (*n* = 162) and randomly matched to individuals with DED but without SjD using the RAND function in Excel to create a balanced dataset. To reduce confounding, only those without other recorded systemic or ocular conditions associated with DED in the clinical protocol (e.g., connective tissue diseases, diabetes mellitus, rosacea, thyroid diseases, psychiatric disorders, atopic diseases, migraine, contact lens use, or prior ocular surgery) were included. This approach aimed to ensure that SjD was the primary distinguishing factor between groups. All DED severity levels were included in both groups.

### Data preparation

Demographic and clinical characteristics were recorded in Excel (Microsoft Corporation, 2018). Features were converted to uniform numerical data of similar grading systems where necessary (e.g., to Meiboscale by Pult et al. [13], rather than percentage dropout). Data preprocessing included handling missing values using mean imputation for continuous variables, encoding categorical variables using one-hot encoding, and deleting variables highly correlated (correlation *≥* 0.8 by Pearson or Spearman tests). The target variable was whether the subject was diagnosed with SjD, binarized as " class 1” and “class 0” for non-SjD; comparison 1:1 matching by age-sex was performed to balance the groups.

The variables were binarized into previously reported thresholds as critical for diagnosing or classifying dry eye severity, including: female sex [15], age group > 50 [15], age group > 70 [15], mild DED symptoms (OSDI 13–22), moderate DED symptoms (OSDI 23–32), severe DED symptoms (OSDI 33–100) [16], blink frequency < 12 blinks/minute [17], blink frequency < 17 blinks/minute [18], osmolarity > 308 mOsm/L, osmolarity > 316 mOsm/L [19], NIBUT < 10 s, NIBUT < 5 s [20], NIBUT < 3 s [21], thin lipid layer < 30 nm (based on Oculus) [11] TMH <2 mm, TMH <1 mm [14], moderate bulbar redness (*≥* 3 based on Oculus) [11], positive InflammaDry test, Schirmer test < 10 mm/5min, Schirmer test <5 mm/5min [22], MG dropout > 75% [13]; cloudy MG expression (grades *≥* 1) [12].

### Statistical analysis

Descriptive statistics were used to summarize categorical variables as counts and frequencies, and continuous variables were expressed as mean and SD. The bivariate analysis involved the Chi-square test for categorical variables, the Student’s t-test for normally distributed quantitative variables with categories, and the Mann-Whitney U test for non-parametric data. A significance threshold was set at *p* < 0.05. Our primary comparison was between the SjD and non-SjD groups, which were evaluated with respect to demographic and clinical parameters.

Symptom–sign discordance was calculated using a methodology adapted from prior studies [7, 23]. The OSDI was used as the symptom parameter, while the OSS, NIBUT, Schirmer test were used as sign parameters. All variables were transformed to a common severity scale ranging from 0 (no symptoms/signs) to 1 (maximum severity) using linear normalization. For variables in which lower values indicate worse disease (NIBUT and Schirmer), values were inverted after normalization to ensure that higher values consistently represented greater disease severity across all parameters.

Discordance scores were calculated as the difference between the normalized OSDI score and each corresponding normalized sign severity score, resulting in values ranging from − 1 to + 1. Positive discordance values indicate a greater symptom burden relative to clinical signs, whereas negative values indicate greater objective disease severity (worse signs) relative to individual-reported symptoms. Values close to zero reflect concordance between symptoms and signs.

After score calculation, individuals were categorized into three groups using predefined cutoffs applied uniformly across all parameters 1) discordant with signs > symptoms (score < − 0.5) 2), concordant (score − 0.5 a + 0.5), and 3) discordant with symptoms > signs (score > + 0.5).

### Machine learning models

The dataset was randomly divided into a training set (70%) and a test set (30%) using the train_test_split function from the scikit-learn library, with a fixed random state (42) to ensure reproducibility. The training set was used to develop and train the machine learning models, while the test set was used exclusively to evaluate their performance. All models were trained and evaluated using the same data split.

Several machine learning models were employed to predict the presence of SjD within the larger DED population. *Logistic regression* is a linear model that estimates the probability of a binary outcome based on input features, using the logistic function to model the probability of the positive class. It is widely used due to its simplicity and interpretability [24]. *Decision tree*, a non-parametric model, makes predictions by recursively partitioning the data into subsets based on feature values, with each internal node corresponding to a feature and the branches representing decision rules that lead to a final prediction at the leaf nodes [25]. *Random forest*, an ensemble learning method, constructs multiple decision trees during training and outputs the class predicted by most trees. This model reduces overfitting and increases prediction accuracy by averaging predictions from diverse decision trees [26].

*Gradient boosting* builds models sequentially, where each new model corrects errors made by the previous models, combining the strengths of weak learners, typically decision trees, to improve overall performance [27]. *Support vector machine* identifies the optimal hyperplane that separates classes in a high-dimensional space and can handle linear and non-linear relationships depending on the kernel used [28]. *K-nearest neighbors* is an instance-based learning algorithm that makes predictions by considering the majority class among the k-nearest training instances to the predicted data point [29]. Lastly, *naive bayes* is a probabilistic classifier based on Bayes’ theorem, assuming independence among predictors and calculating the posterior probability for each class based on the input features [30].

**Fig. 1:**
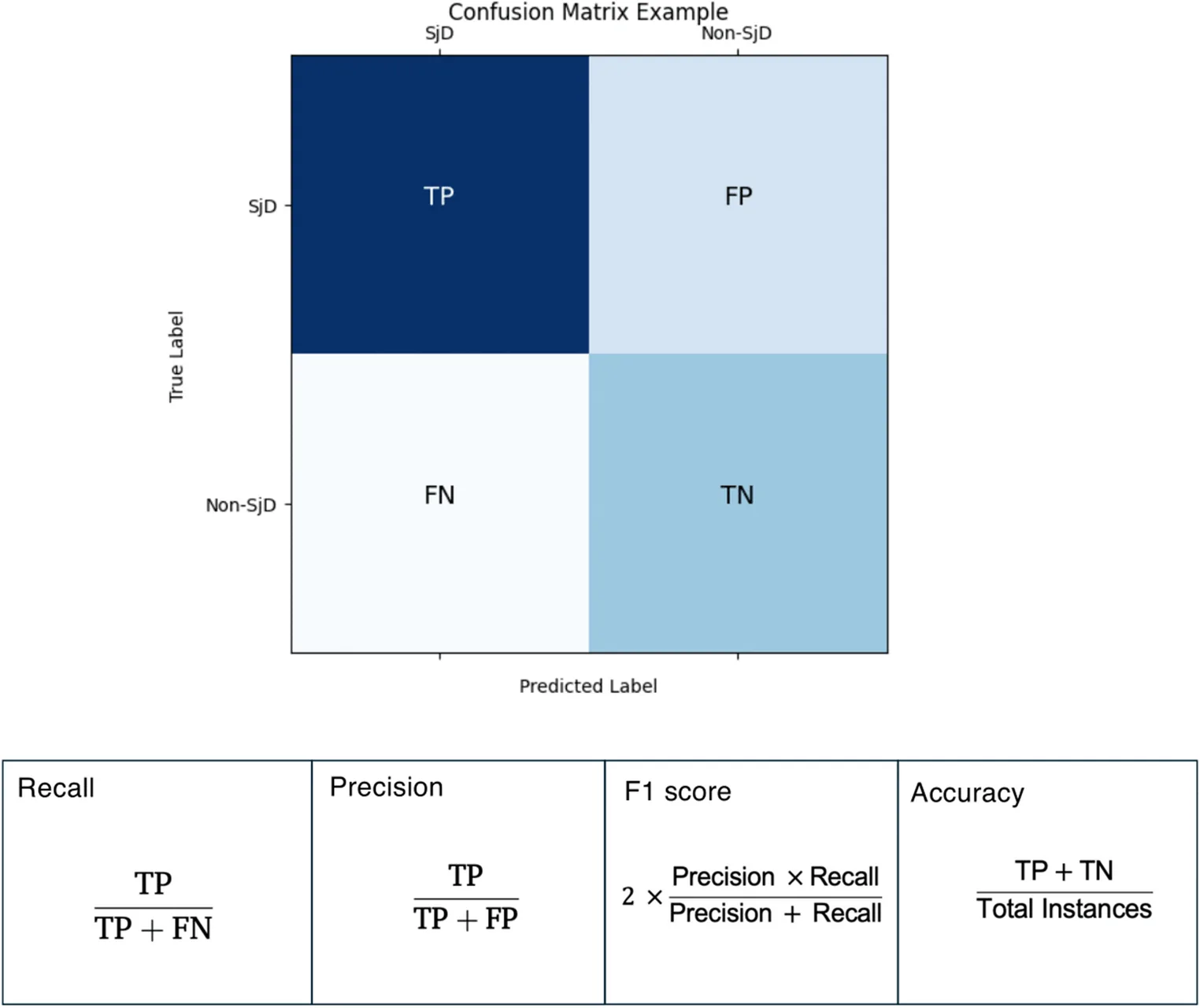
Metric for binary classification task included. TP: True positive, TN: True negative, FP: False positive, FN: False negative Recall: Measures the ability to find all positive cases. Precision: Measures the accuracy of positive predictions. Accuracy: Measures how often the model is correct overall. Use when classes are balanced. F1 Score: Combines precision and recall into a single measure. Use when you need a balance between precision and recall, especially with imbalanced data

To evaluate the algorithms’ performance, we used a set of metrics chosen based on insights obtained from Hicks et al. [31]. Specifically, we used recall, precision, F-1 score, and accuracy. An explanation of each metric is provided in Fig. 1. All performance metrics reported in this study correspond to the test dataset. The “optimal” model was selected based on the model with higher accuracy in the test dataset.

In the optimal model, feature importance and mutual information gain were calculated. These metrics indicate the relative contribution of each feature to the model’s predictive performance and the amount of shared information between the feature and the target variable, respectively [32]. It is important to note that feature importance indicates how much a variable contributes to distinguishing classes but does not specify which category (e.g., positive or negative InflammaDry test result) is related to SjD or non-SjD.

To determine the direction and strength of these associations, the Weight of Evidence (WoE) and Information Value (IV) were calculated for the top five variables identified in the optimal model. WoE is a tool for assessing the strength and direction of the relationship between predictor variables and the binary outcome (in this case, SjD versus non-SjD DED) [33]. Positive WoE values indicate that a characteristic is positively associated with the event, such as the presence of SjD, whereas negative WoE values suggest an inverse relationship [33]. The magnitude of WoE further provides information about the strength of the predictor. A WoE value near zero indicates little or no predictive power, while larger absolute WoE values—whether positive or negative—indicate stronger relationships between the predictor and the outcome [33]. The IV quantifies the predictive strength of each feature within the model. IV values are interpreted based on established thresholds: values < 0.02 indicate no predictive power, 0.02–0.1 are considered weak, 0.1–0.3 moderate, > 0.3–0.5 strong, and values > 0.5 reflect very strong predictive power [33].

### Ethics statement

Approval from the Universidad del Rosario Institutional Review Board was obtained (DVO005 2208–CV1662) to review charts and analyze the database. Additionally, this study was conducted in adherence to the Declaration of Helsinki.

## Results

Three hundred twenty-four subjects with DED were analyzed including 162 individuals with SjD and 162 individuals without SjD. 266 (82.1%) were female, and 58 (17.9%) were male. The mean age was 47.4 ± 15.71 years; demographics were similar across the groups.

Regarding symptoms, the SjD group had lower OSDI scores compared to the non-SjD group (31.8 vs. 44.6, *p* < 0.001). In contrast, objective ocular surface parameters were generally worse in the SjD group. Specifically, higher OSS: with a greater proportion of individuals classified as Oxford III (26 [16.0%] vs. 11 [6.7%]) and Oxford IV (9 [5.5%] vs. 8 [4.9%], *p* = 0.015), lower NIBUT (8.66 vs. 10.60 s, *p* < 0.001), lower Schirmer test results (7.5 vs. 14.4 mm, *p* = 0.001), lower TMH (0.21 vs. 0.23 mm, *p* = 0.033), higher nasal (1.76 vs. 1.28, *p* < 0.001) and temporal (1.44 vs. 1.28, *p* = 0.003) bulbar redness and a higher frequency of InflammaDry positivity (73.4% versus 25.3%; *p* < 0.001), suggesting discordance of subject-reported symptoms and objective clinical signs, with the SjD group exhibiting more severe ocular surface signs despite reporting fewer symptoms (Table 1).

**Table 1:** Demographic and clinical characteristics

	Total (*N* = 324)	With SjD (*N* = 162)	Without SjD (*N* = 162)	*p* Value
Demographics				
**Sex**				0.854
Female	266 (82.1%)	135 (83.4%)	131(80.9%)	
Male	58 (17.9%)	27 (16.6%)	31 (19.1%)	
**Age**	47.4 ± 15.71	47.2 ± 16.11	48.1 ± 13.80	0.755
**Signs and symptoms**				
** OSDI**	38.2 ± 18.82	31.8 ± 19.10	44.6 ± 16.10	**< 0.001**
**OSS with fluorescein staining**				**0.015**
Oxford 0	39 (12.0%)	12 (7.4%)	27 (16.6%)	
Oxford I	152 (46.9%)	65 (40.1%)	87 (53.7%)	
Oxford II	79 (24.4%)	50 (30.8%)	29 (17.9%)	
Oxford III	37 (11.3%)	26 (16.0%)	11 (6.7%)	
Oxford IV	17 (5.2%)	9 (5.5%)	8 (4.9%)	
**NIBUT (seconds)**	9.6 ± 5.20	8.6 ± 5.07	10.6 ± 5.24	**< 0.001**
**Schirmer test with anesthesia (mm/5min)**	10.9 ± 8.48	7.5 ± 6.86	14.4 ± 10.10	**< 0.001**
**TMH (mm)**	0.22 ± 0.11	0.2 ± 0.10	0.2 ± 0.12	**0.033**
**Nasal bulbar redness (Oculus score)**	1.5 ± 0.60	1.7 ± 0.71	1.2 ± 0.43	**< 0.001**
**Temporal ocular redness (Oculus score)**	1.3 ± 0.40	1.4 ± 0.51	1.2 ± 0.47	**0.003**
**Positive InflammaDry test**	160 (49.4%)	119 (73.4%)	41 (25.3%)	**< 0.001**
**Osmolarity (mOsm/L)**	301 ± 10.80	300 ± 8.62	303 ± 12.50	**0.024**
**Superior MG dropout**				**< 0.001**
0%	72 (22.2%)	41 (25.3%)	31 (19.1%)	
1–24%	119 (36.7%)	80 (49.3%)	39 (24.0%)	
25–50%	68 (21.0%)	23 (14.1%)	45 (27.7%)	
51–75%	42 (13.0%)	2 (1.2%)	40 (24.6%)	
> 75%	23 (7.1%)	16 (9.8%)	7 (4.3%)	
**Inferior MG dropout**				**< 0.001**
0%	51 (15.7%)	22 (13.5%)	29 (17.9%)	
1–24%	127 (39.2%)	81 (50.0%)	46 (28.3%)	
25–50%	86 (26.5%)	46 (28.3%)	40 (24.6%)	
51–75%	50 (15.4%)	11 (6.7%)	39 (24.0%)	
> 75%	10 (3.1%)	2 (1.2%)	8 (4.9%)	
**Thin Lipid layer (< 30 nm)**	245 (75.6%)	128 (79.0%)	117 (72.2%)	0.155
**MG expressibility**		**0.016**		
Clear	175 (40.7%)	97 (59.8%)	78 (48.1%)	
Cloudy with mild pressure	93 (28.7%)	55 (33.9%)	38 (23.4%)	
Cloudy with moderate pressure	51 (15.7%)	24 (14.8%)	27 (16.6%)	
No expression with hard pressure	5 (1.5%)	5 (3.0%)	0 (0%)	
**Blink frequency (blinks per minute)**,	16.9 ± 10.8	14.5 ± 9.52	15.1 ± 11.60	0.591
**Signs-symptoms discordance score**				
**OSS discordance**	−0.13 ± 0,30	−0.34 ± 0.29	0.09 ± 0.30	**< 0.001**
**OSS discordance by group**				
Concordant (score − 0.5 a + 0.5)	235 (72.5%)	110 (67.9)	125 (77.2%)	**0.002**
Discordant with symptoms > signs (score > + 0.5)	32 (9.9%)	12 (7.4%)	20 (12.3%)	
Discordant with signs > symptoms (score < − 0.5)	57 (17.6%)	40 (24.7%)	17 (10.5%)	
**NIBUT discordance**	−0.50 ± 0,24	−0.60 ± 0.25	−0.39 ± 0.22	**< 0.001**
**NIBUT discordance by group**				
Concordant (score − 0.5 a + 0.5)	156 (48.1%)	43 (25.8%)	113 (69.8%)	**0.001**
Discordant with symptoms > signs (score > + 0.5)	6 (1.9%)	2 (1.2%)	4 (2.4%)	
Discordant with signs > symptoms (score < − 0.5)	162 (50%)	117 (72.2%)	45 (27.8%)	
**Schirmer discordance**	−0.43 ± 0,33	−0.46 ± 0.31	−0.39 ± 0.34	**0.003**
**Schirmer discordance by group**				
Concordant (score − 0.5 a + 0.5)	156 (48.1%)	70 (43.2%)	86 (53.1)	**0.002**
Discordant with symptoms > signs (score > + 0.5)	14 (4.3%)	5 (3.1%)	9 (5.5%)	
Discordant with signs > symptoms (score < − 0.5)	144 (44.4%)	87 (53.7)	57 (35.1%)	

*SjD *Sjögren’s disease, *SD *Standard deviation, *OSDI *Ocular Surface Disease Index, *OSS *Ocular Surface Staining, *MG *Meibomian gland, *TMH *Tear Meniscus Height, *NIBUT. *Non-invasive tear break-up time Variables shown in bold represent the main parameters evaluated, whereas non-bold entries correspond to their respective subcategories

Consistent with this, discordance scores differed significantly between groups. For all evaluated parameters (OSS, NIBUT, and Schirmer), mean discordance scores were more negative in the SjD group compared to the non-SjD group (OSS: −0.34 ± 0.29 vs. +0.09 ± 0.30, *p* < 0.001; NIBUT: −0.60 ± 0.25 vs. −0.39 ± 0.22, *p* < 0.001; Schirmer: −0.46 ± 0.31 vs. −0.39 ± 0.34, *p* = 0.003), indicating a consistent predominance of clinical signs over symptoms in SjD. This pattern was further supported by categorical analysis, as a higher proportion of individuals with SjD were classified as discordant with signs > symptoms (score < − 0.5) for OSS (24.7% vs. 10.5%), NIBUT (72.2% vs. 27.8%), and Schirmer test (53.7% vs. 35.1%, *p* = 0.002). Taken together, these findings indicate that SjD-related DED is consistently characterized by greater objective disease severity relative to subject-reported symptoms across multiple ocular surface parameters (Table 1).

The final dataset for the machine learning models consisted of 37 features of these 324 subjects. Missing data were found in the following features: age (five records, 1.5%), bulbar redness (nine records, 2.8%), and Schirmer (eight records, 2.5%). Given the low proportion of missing values (< 5% for all variables), mean imputation was applied.

The logistic regression model achieved an accuracy of 93%, with a weighted average recall, precision, and F1 score of 0.94. This indicates that out of 100 cases, the model correctly identified 93 as either SjD or non-SjD. Using a decision tree model, overall accuracy remained at 93%, with a slight improvement in precision for class 1 (0.98 vs. 0.96 with logistic regression), indicating improved identification of SjD cases. The weighted average recall, precision, and F1 Score of logistic regression and decision tree models were 0.94, suggesting that both models effectively handled the classification across classes.

The random forest model increased accuracy to 95%, with weighted average precision and recall reaching 0.96. This highlights the model’s ability to balance sensitivity and specificity across all classes. The F1 score was also 0.96, meaning that the model maintained a strong equilibrium between its ability to correctly identify true SjD cases and avoid incorrectly labeling non-SjD cases as positive. Other models, such as support vector machine and k-nearest neighbors, demonstrated lower performance, achieving 92% and 89% accuracy, respectively. Naive bayes exhibited the poorest performance, with an accuracy of 53%, emphasizing its limitations in recognizing the distinct features of SjD-related-DED.

The gradient boosting model was the best-performing approach, achieving an accuracy of 96% and a weighted average precision, recall, and F1 score of 0.97. These results indicate strong performance in identifying true SjD cases (high recall) while minimizing false positives (high precision), with the F1 score reflecting a balanced performance across both classes. (Table 2).

**Table 2:** Machine learning models results

Model	Recall	Precision	F1-Score	Accuracy
Logistic Regression				0.93
DED without SjD	0.96	0.92	0.94	
DED with SjD	0.91	0.96	0.93	
Weighted average	0.94	0.94	0.94	
Decision Tree				0.93
DED without SjD	0.98	0.91	0.94	
DED with SjD	0.89	0.98	0.93	
Weighted average	0.94	0.94	0.94	
Random Forest				0.95
DED without SjD	0.98	0.94	0.96	
DED with SjD	0.94	0.98	0.96	
Weighted average	0.96	0.96	0.96	
Gradient Boosting				0.96
DED without SjD	0.98	0.96	0.97	
DED with SjD	0.96	0.98	0.97	
Weighted average	0.97	0.97	0.97	
Support Vector Machine				0.92
DED without SjD	0.96	0.91	0.93	
DED with SjD	0.89	0.95	0.92	
Weighted average	0.93	0.93	0.93	
K-Nearest Neighbors				0.89
DED without SjD	0.84	0.96	0.90	
DED with SjD	0.96	0.85	0.90	
Weighted average	0.90	0.90	0.90	
Naive Bayes				0.53
DED without SjD	1.00	0.53	0.69	
DED with SjD	0.02	1.00	0.04	
Weighted average	0.53	0.75	0.38	

*DED *Dry eye disease, *SjD *Sjögren’s disease

**Fig. 2:**
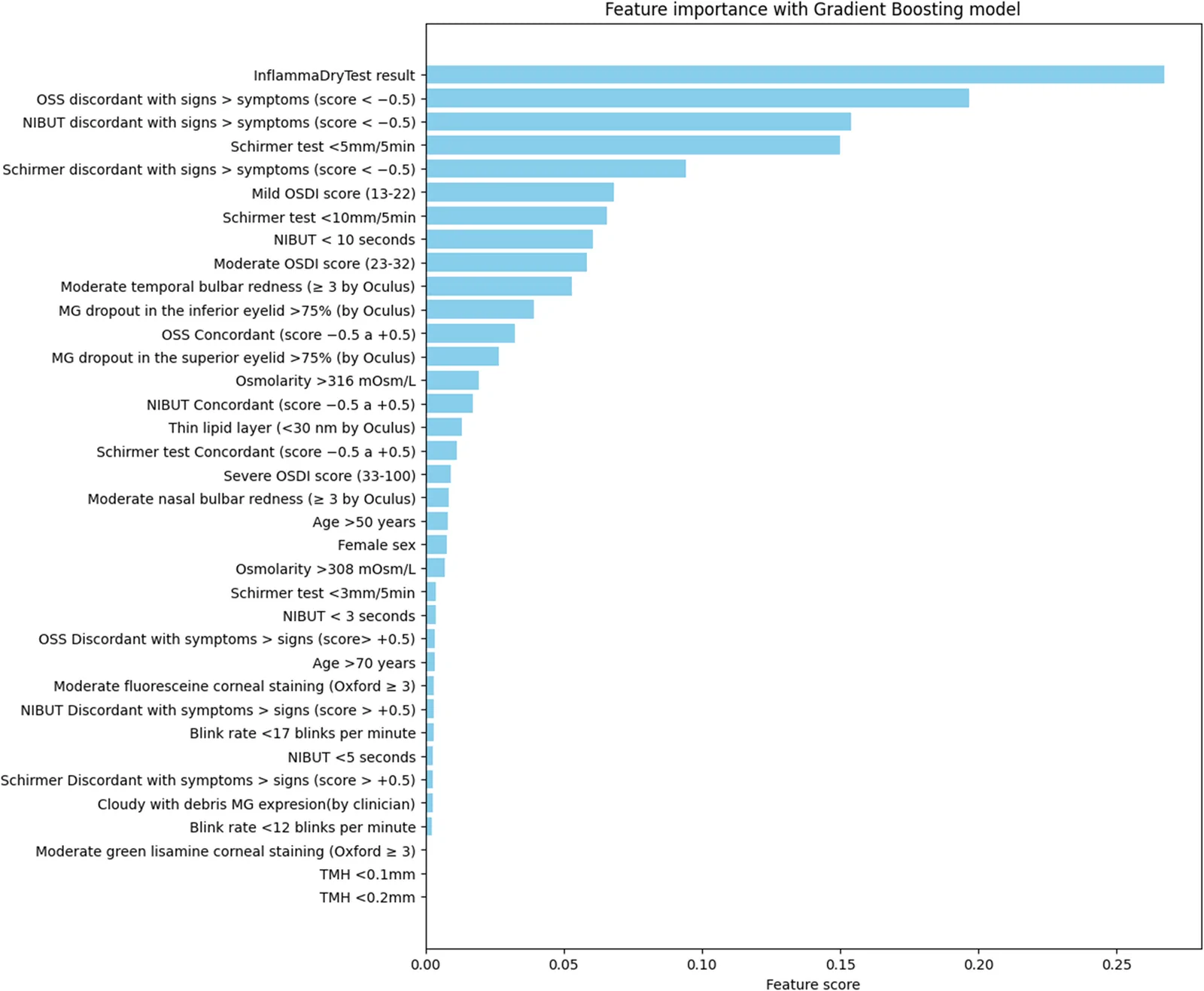
Feature of importance to distinguish dry eye disease with and without Sjögren’s disease using the Gradient Boosting model. OSDI: Ocular Surface Index score, OSS: Ocular surface staining, NIBUT: Non-invasive tear break-up time, MG: Meibomian gland, TMH: Tear meniscus height

Examining feature importance in the optimal model (gradient boosting), the five most influential variables were a positive InflammaDry result (rank #1; importance = 0.267), OSS discordant with signs > symptoms (score < − 0.5) (rank #2; importance = 0.196), NIBUT discordant with signs > symptoms (score < − 0.5); (rank #3; importance = 0.153), a Schirmer test < 5 mm/5 min (rank #4; importance = 0.149) and Schirmer discordant with signs > symptoms (score < − 0.5) (rank #5; importance = 0.094). These findings highlight the InflammaDry test, discordant with signs > symptoms, and the Schirmer test as key features for differentiating SjD from non-SjD DED (Fig. 2 and Supplementary Material 2).

**Fig. 3:**
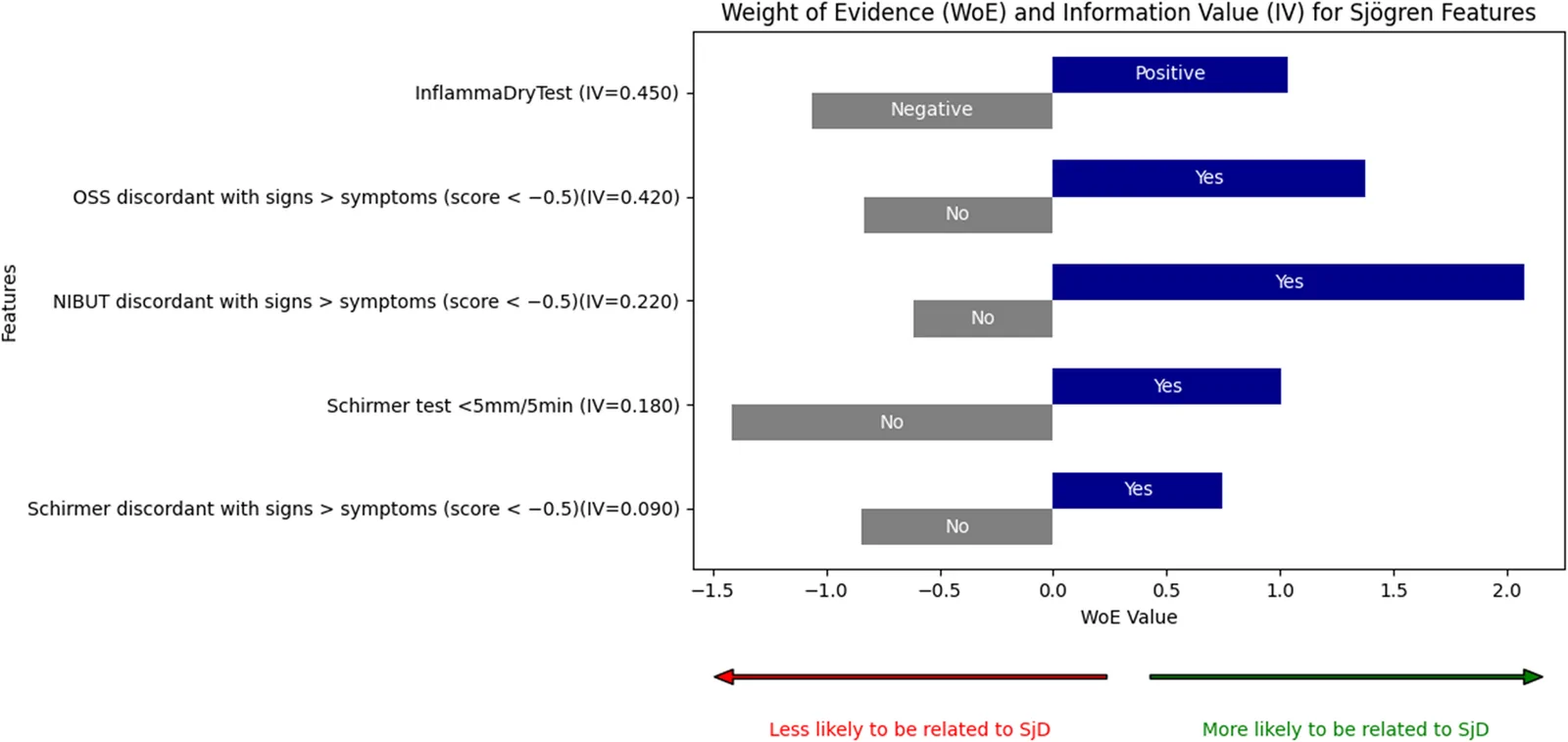
Weight of Evidence and Information Value for Sjögren’s top five Features in the Gradient Boosting model. SjD: Sjögren’s disease, WoE: Weight of evidence, IV: Information value, OSS: Ocular surface staining, NIBUT: Non-invasive tear break-up time

Examining WoE in the five most influential variables further improved the identification of whether the presence of these features was associated with SjD or non-SjD. All variables demonstrated positive WoE values: positive InflammaDry test (WoE: 1.034), OSS discordant with signs > symptoms (score < − 0.5) (WoE: 1.376), NIBUT discordant with signs > symptoms (score < − 0.5) (WoE: 2.079), Schirmer test < 5 mm/5 min (WoE: 1.007), Schirmer discordant with signs > symptoms (score < − 0.5) (WoE: 0.746). Indicating that the presence of these features is more strongly associated with SjD (as opposed to non-SjD), as reflected by positive values (Fig. 3).

Information Value further supported the predictive strength of these features. The strongest predictors were positive InflammaDry test (IV: 0.450) and OSS discordant with signs > symptoms (score < − 0.5) (IV: 0.420), both demonstrating strong discriminatory power. Moderate predictive ability was observed for NIBUT discordant with signs > symptoms (score < − 0.5) (IV: 0.220) and Schirmer test < 5 mm/5 min (IV: 0.180). Schirmer discordant with signs > symptoms (score < − 0.5) showed weaker predictive value (IV: 0.090). Overall, these results support that combining inflammatory markers with sign-dominant discordance patterns provides discriminatory power in identifying SjD-related DED (Fig. 3).

## Discussion

In this study, we evaluated the potential of machine learning models to differentiate SjD from non-SjD DED, using exclusively ocular data. Among the models tested, gradient boosting demonstrated the highest performance, achieving an accuracy of 0.96. The most influential features associated with SjD-related DED included a positive InflammaDry test, discordant pattern with signs > symptoms (score < − 0.5) for OSS, NIBUT, and Schirmer, and a Schirmer test < 5 mm/5 min. These findings highlight the potential of data-driven approaches to distinguish SjD-related DED from other causes based solely on ocular surface characteristics. Notably, two core clinical elements emerged as central to SjD-related DED: the presence of ocular surface inflammation, measured by a positive InflammaDry test, and a symptom-sign discordance pattern characterized by worse signs than symptoms.

Prior studies have also found that individuals with SjD have a higher burden of ocular surface inflammation compared to their non-SjD DED counterparts, including using the InflammaDry test. In a study of 21 individuals with SjD-related DED and 20 with non-SjD DED, MMP-9 positivity was higher in the SjD group (65% versus 24%; OR = 5.8, 95% CI: 4.46–19.81, *p* < 0.05) [34]. Similarly, in a cohort of 1,363 eyes, a positive InflammaDry test was 1.59 times more likely in individuals with autoimmune or allergic disease, including SjD (OR = 1.59, 95% CI: 1.19–2.13, *p* = 0.002) [35]. These findings support the concept that increased MMP-9 expression, as indicated by a positive InflammaDry result, is common in SjD-related DED; consistent with this, InflammaDry positivity emerged as the most influential variable distinguishing SjD-related from non-SjD DED. Beyond MMP-9, other inflammatory mediators are expressed at higher levels in SjD. In 10 individuals with SjD compared to 10 controls, higher mRNA levels of IL-1α (4.32 ± 3.6 vs. 0.88 ± 1.2 gene/mimic ratio, *p* = 0.003), IL-6 (3.69 ± 3.2 vs. 0.29 ± 0.5 gene/mimic ratio, *p* < 0.001), IL-8 (2.2 ± 1.6 vs. 0.82 ± 0.3 gene/mimic ratio, *p* < 0.001), and TNF-α (4.15 ± 4.7 vs. 1.2 ± 0.9 gene/mimic ratio, *p* = 0.015), captured using conjunctival impression cytology, were noted in cases [36]. Although no cytokines were included in our machine learning models, these data highlight that elevated inflammatory burden is characteristic of SjD-related DED and supports the potential integration of additional ocular inflammatory biomarkers in future models to further improve disease discrimination.

Similarly, previous studies have documented worse clinical signs compared to symptoms in SjD. For instance, one study found that 55 individuals with 2016 ACR/EULAR–defined SjD had significantly lower OSDI scores compared to 221 subjects with DED but no SjD (36.3 ± 20.5 vs. 43.6 ± 21.1, *p* = 0.022), despite exhibiting more severe DED signs. Specifically, individuals with SjD demonstrated reduced tear secretion (4.9 ± 2.5 vs. 9.8 ± 6.9 mm, *p* = 0.001) and higher OSS (3.2 ± 2.3 vs. 1.7 ± 2.0, *p* = 0.016), indicating greater ocular surface damage despite reporting fewer symptoms [8]. A multicenter study of 535 individuals with moderate to severe DED further quantified this relationship using discordance scores, with SjD associated with sign-dominant discordance (β = − 0.175, *p* = 0.002). Supporting this, correlations between subjective symptoms and objective signs were consistently weak (e.g., corneal staining ρ = 0.12, *p* = 0.006; TBUT ρ = − 0.05, *p* = 0.25), underscoring the limited alignment between subject-reported symptoms and clinical findings [37]. Taken together, these data support the concept that SjD-related DED is characterized by a mismatch between symptoms and signs, with individuals reporting fewer symptoms than expected for the degree of ocular surface damage. The gradient boosting model findings reinforce this, demonstrating a discordant pattern of signs > symptoms (score < − 0.5) for OSS, NIBUT, and Schirmer among the top five predictive features for differentiating SjD-related DED from non-SjD DED.

This dissociation is thought to result from structural and functional corneal innervation alterations in SjD [38–41]. For instance, in 35 individuals with SjD and 20 controls, in vivo confocal microscopy (IVCM) images demonstrated reduced sub-basal nerve density in cases (3.34 vs. 5.10 nerves/frame, *p* < 0.0001) [42]. In fact, individuals with SjD show lower sub-basal nerve density not only when compared with controls (2.9 vs. 5.8 nerves/frame, *p* < 0.001) but also compared to individuals with non-SjD related DED (2.9 vs. 3.9 nerves/frame, *p* < 0.001) [43], underscoring that abnormal corneal nerve anatomy is a feature of SjD-related DED. Functionally, reduced corneal sensitivity has also been demonstrated in SjD. One study using the Belmonte non-contact esthesiometer found that individuals with SjD had significantly higher mechanical detection thresholds (147 ± 21 mL/min) than both younger (78 ± 12 mL/min, *p* < 0.001) and older (106 ± 21 mL/min, *p* = 0.024) controls, indicating decreased sensitivity [41].

It is important to recognize, however, that not all individuals with SjD demonstrate a corneal neuropathy and that neuropathic contributors have also been found in a subset of individuals [44]. For example, in 108 individuals with SjD, seronegative subjects reported higher overall pain severity compared to seropositive subjects (4.70 ± 1.92 vs. 3.45 ± 2.25, *p* = 0.003), and also reported greater pain interference with daily activities (4.72 vs. 3.33, *p* = 0.01), despite similar or less pronounced objective signs of gland dysfunction [45]. This pattern contrasts with our findings, demonstrating that some individuals with SjD exhibit symptoms that are disproportionate to their clinical signs. This phenotype is supported by the heterogeneity observed in structural and functional corneal nerve findings in SjD, with some studies noting similar nerve densities across SjD and control groups (5.9 vs. 6.1 nerves/frame, *p* = 0.78), but higher corneal sensitivity in SjD (54.5 vs. 85.0 mL/min, *p* = 0.04) [46]. Furthermore, negatively correlation between corneal sensitivity and both ocular surface staining (*r* = − 0.51, *p* < 0.05) and symptom scores (*r* = − 0.46, *p* < 0.01), supports an SjD phenotype in which corneal nerve hypersensitivity is associated with symptom–sign discordance. It is interesting that a pattern of symptoms exceeding signs was not identified as a distinguishing feature of SjD-related DED in our machine learning model. Several factors may explain this finding. First, neuropathic pain questionnaires, IVCM, and sensitivity testing were not included in the clinical protocol, potentially limiting the identification of individuals with a neuropathic symptom profile. Second, as our referral center focuses on detailed ocular surface characterization, individuals are more likely to be referred when objective signs are pronounced, whereas individuals with milder or symptom-predominant disease may be managed empirically without advanced diagnostic testing, introducing a potential selection bias.

This study has some limitations. First, the retrospective, single-center design and relatively small sample size may limit generalizability. Although the dataset was split into training (70%) and test (30%) sets to evaluate model performance, no independent external validation was used. Therefore, further validation in larger, independent datasets is needed to confirm the reproducibility of these findings. Second, the Schirmer test was performed with anesthesia and after slit-lamp examination, which may have influenced tear secretion; accordingly, the test order should be standardized. Third, potential confounders, including topical and systemic treatments (e.g., artificial tears, anti-inflammatory drops, or oral secretagogues), menopausal status, systemic medication use, and psychiatric comorbidities, were not systematically captured and may have affected symptom perception and/or ocular surface parameters. Finally, the diagnosis of SjD was established externally, precluding verification of the diagnostic criteria used. Incorporating objective diagnostic markers such as anti-Ro/SSA or anti-La/SSB serology and minor salivary gland biopsy could strengthen future models by enabling direct comparison with established classification criteria. Overall, these findings require validation in larger, prospective, and ethnically diverse cohorts.

In summary, machine learning models were able to distinguish SjD-related from non-SjD DED using non-invasive clinical data with high accuracy. Individuals with SjD more frequently exhibit ocular surface inflammation, as reflected by InflammaDry positivity, along with a consistent pattern of symptom–sign discordance characterized by more severe clinical signs relative to reported symptoms. Among objective measures, a Schirmer test < 5 mm/5 min emerged as the only independent clinical sign associated with SjD, reinforcing its relevance within current diagnostic frameworks. The features identified in this study may serve as the basis for developing automated screening tools in ophthalmology and rheumatology settings, potentially enabling earlier recognition of SjD-related DED in high-risk populations. However, further refinement and external validation of these models are required before their implementation in routine clinical practice.

## Supplementary Information

Supplementary Material 1.

Supplementary Material 2.

## Data Availability

The corresponding author can share information in the databases used in this article upon reasonable request.
