# Supplementary material for: Which features best differentiate Sjögren’s-related from non-Sjögren’s-related dry eye disease?
Source: Graefes Arch Clin Exp Ophthalmol. 2026 Jul 8;264(11):3417–28. doi: 10.1007/s00417-026-07334-w (PMC13736476; doi:10.1007/s00417-026-07334-w)
Supplement: Supplementary file 1 [file 417_2026_7334_MOESM1_ESM.docx]

**Supplementary Material 1: Clinical Protocol**

**Determination of non-invasive tear break-up time**

The non-invasive tear break-up time (NIBUT) was measured using the Oculus Keratograph 5M. This technique involves projecting a Placido ring onto the ocular surface and focusing it clearly. After the subject completes one blink, they are instructed to stop blinking. The Oculus Keratograph 5M automatically detects the first break-up detected in any surface segment (1). A NIBUT <10 seconds was considered abnormal, while values ≥10 seconds were considered normal (2).

**Lipid layer thickness**

The lipid layer thickness was obtained using Oculus Keratograph 5M. The subjects were instructed to focus on the camera, which recorded a 20-second video of the tear film. The lipid layer thickness was considered thin when it was lower than 30 nm; values ≥ 30 nm were considered normal (1).

**Determination of Osmolarity**

Tear osmolarity was measured using the Tear Lab Osmolarity System. The tear collection procedure followed the user manual instructions. (3) The patient was seated with their head tilted upwards and eyes looking up. The tip of the osmometer was positioned just above the lower lid in the lateral canthal area until it touched the thin line of the tear film. A beeping sound and the disappearance of the green light confirmed successful tear collection. The osmometer was then placed into the reader within 5–10 seconds, the test card code was selected, the OK button was pressed, and the test result was obtained (3). An osmolarity >308 mOsm/L in either eye or an interocular difference >8 mOsm/L was considered abnormal (2).

**Determination of ocular surface staining.**

For fluorescein staining, a picture was taken two minutes after instilling a single drop of 0.25% fluorescein sodium in each eye, with a cobalt blue filter in the slit lamp. For lissamine green staining, a single drop of 1% lissamine green solution was applied to each eye, and the subjects were instructed to blink gently. Two minutes after instillation, a picture was taken and analyzed using a red-free filter in the slit lamp (1). Staining severity was graded from 0 to 5 based on a comparison of the captured photographs with the standardized reference images published by Bron et al.(4), as one of the grading systems recommended by Dry Eye Workshop-II (2) and the American Academy of Ophthalmology (5).

**Determination of ocular redness**

Conjunctival redness was assessed using a photograph taken with the Topcon DC3 camera. The image of the exposed bulbar conjunctiva was then scanned and analyzed by the Oculus Keratograph 5M, automatically generating a score based on the Jenvis Classification. This classification system evaluates the area percentage ratio between blood vessels and the rest of the scanned bulbar conjunctiva, providing a score on a scale from 0 to 4: normal (0-0.9), mild (1-1.9), moderate (2-2.9), and severe (3-4). The Oculus provides two distinct measurements, one for the nasal region and one for the temporal region (1,6).

**Determination of Meibography**

Meibography was performed using the Oculus Keratograph 5M, based on the criteria proposed by Pult and Riede-Pult (7). The evaluation defined the area of Meibomian gland loss (MGL) as the percentage of the area without visible glands in relation to the total visible tarsal area, assigning a score from 0 to 4. Both the upper and lower eyelid Meibomian glands were visualized and graded. A score of 0 represented no atrophy; a score of 1 indicated ≤25% MGL; a score of 2 indicated 26% to 50% MGL; a score of 3 indicated 51% to 75% MGL; and a score of 4 indicated >75% MGL (8).

**InflammaDry Testing**

For the InflammaDry test (Inoftal, Quidel Corporation, USA), the operator collected tear samples by dabbing the patient's lower palpebral conjunctiva 6–8 times with the sample collector without using anesthetic or fluorescein to achieve saturation indicated by a pink or glistening fleece. The saturated fleece was inserted into the cassette, and the tip was immersed in a buffering solution for 20 seconds. Results were determined after 10 minutes, with one blue and one red line indicating a positive result (MMP-9 ≥40 ng/mL) and a single blue line a negative result (MMP-9 <40 ng/mL). InflammaDry positivity was determined at the clinician's discretion.

**Schirmer test**

The Schirmer I test with anesthesia was performed after the InflammaDry test by applying a drop of proparacaine hydrochloride 0.5% in each eye and placing a Schirmer strip in the inferior conjunctival fornix. The measured length of wetting after a 5-minute period was recorded (9). A result of <10 mm was considered abnormal (2).

**Corneal sensitivity determination**

Corneal sensitivity was evaluated with the Cochet-Bonnet esthesiometer (Luneau Ophtalmologie, Chartres, France). It provides repetitive information about corneal sensitivity through subjective patient responses. It consists of a nylon monofilament with a diameter of 0.12 mm that can be modified in length from 0 to 60 mm to obtain a different pressure applied to the cornea. It produces a pressure ranging from 0.96 to 17.68 g/mm2; a 60-mm length corresponds to 0.96 g/mm2, a 55-mm length to 1.08 g/mm2, and so on; finally, a 5-mm length corresponds to 17.68 g/mm2, according to the manufacturer’s instructions (10). A range from 0.4g/mm2 to 0.8g/mm2 was considered normal (11).

**References**

1.	Kunert K, Sickenberger W. Dry Eye Guide: A Guide to Comprehensive Dry Eye Screening with the OCULUS Keratograph 5M [Internet]. 2017. Disponible en: https://d3ciwvs59ifrt8.cloudfront.net/7c82a155-a2d2-4de8-a159-f406905e28ca/fa6699ea-8ed7-4c98-ac39-b39186a7c94e.pdf

2.	Wolffsohn JS, Arita R, Chalmers R, Djalilian A, Dogru M, Dumbleton K, et al. TFOS DEWS II Diagnostic Methodology report. The Ocular Surface. 2017;15(3):539-74.

3.	TearLab. TearLab Osmolarity System User Manual [Internet]. 2012. Disponible en: https://trukera.com/wp-content/uploads/2022/08/TearLab_Osmolarity_System_User_Manual_-_English.pdf

4.	Bron AJ, Evans VE, Smith JA. Grading Of Corneal and Conjunctival Staining in the Context of Other Dry Eye Tests: Cornea. 2003;22(7):640-50.

5.	American Academy of Ophthalmology. Oxford Grading System [Internet]. Disponible en: https://www.aao.org/education/image/oxford-grading-system

6.	Di Cello L, Pellegrini M, Vagge A, Borselli M, Ferro Desideri L, Scorcia V, et al. Advances in the Noninvasive Diagnosis of Dry Eye Disease. Appl Sci. 2021;11(21):10384.

7.	Pult H, Riede-Pult B. Comparison of subjective grading and objective assessment in meibography. Cont Lens Anterior Eye. 2013;36(1):22-7.

8.	Robin M, Liang H, Rabut G, Augstburger E, Baudouin C, Labbé A. The Role of Meibography in the Diagnosis of Meibomian Gland Dysfunction in Ocular Surface Diseases. Transl Vis Sci Technol. 2019;8(6):6.

9.	Senchyna M, Wax MB. Quantitative assessment of tear production: A review of methods and utility in dry eye drug discovery. j ocul biol dis inform. marzo de 2008;1(1):1-6.

10.	Roszkowska AM, Colosi P, Ferreri FMB, Galasso S. Age-Related Modifications of Corneal Sensitivity. Ophthalmologica. 2004;218(5):350-5.

11.	McDonnell C, Boland C, Khalid K, Osman M. Corneal sensitivity in normal subjects as measured using the Cochet-Bonnet aesthesiometer [Internet]. Technological University Dublin; 2023 [citado 10 de noviembre de 2024]. Disponible en: https://arrow.tudublin.ie/scschphycon/32/
