# Supplementary material for: Which features best differentiate Sjögren’s-related from non-Sjögren’s-related dry eye disease?
Source: Graefes Arch Clin Exp Ophthalmol. 2026 Jul 8;264(11):3417–28. doi: 10.1007/s00417-026-07334-w (PMC13736476; doi:10.1007/s00417-026-07334-w)
Supplement: Supplementary file 2 [file 417_2026_7334_MOESM2_ESM.docx]

**Supplementary material 2: Feature importance and mutual information gain using the Gradient Boosting model**

| **Feature Description** | **Importance** | **Mutual Information Gain** |
| --- | --- | --- |
| InflammaDry test result | 0.2671 | 0.1163 |
| OSS discordant with signs > symptoms (score < −0.5) | 0.1965 | 0.1201 |
| NIBUT discordant with signs > symptoms (score < −0.5) | 0.1539 | 0.1339 |
| Schirmer test <5 mm/5 min | 0.1499 | 0.1184 |
| Schirmer discordant with signs > symptoms (score < −0.5) | 0.0943 | 0.1211 |
| Mild OSDI score (13–22) | 0.0681 | 0.1263 |
| Schirmer test <10 mm/5 min | 0.0658 | 0.0497 |
| NIBUT <10 seconds | 0.0605 | 0.0542 |
| Moderate OSDI score (23–32) | 0.0583 | 0.0002 |
| Moderate temporal bulbar redness (≥3 by Oculus) | 0.0528 | 0.0511 |
| MG dropout in the inferior eyelid >75% (by Oculus) | 0.0391 | 0.0478 |
| OSS concordant (score −0.5 to +0.5) | 0.0324 | 0.0030 |
| MG dropout in the superior eyelid >75% (by Oculus) | 0.0264 | 0.0513 |
| Osmolarity >316 mOsm/L | 0.0192 | 0.0042 |
| NIBUT concordant (score −0.5 to +0.5) | 0.0173 | 0.0093 |
| Thin lipid layer (<30 nm by Oculus) | 0.0133 | 0.0000 |
| Schirmer concordant (score −0.5 to +0.5) | 0.0113 | 0.0172 |
| Severe OSDI score (33–100) | 0.0092 | 0.1263 |
| Moderate nasal bulbar redness (≥3 by Oculus) | 0.0083 | 0.0398 |
| Age >50 years | 0.0082 | 0.0000 |
| Female sex | 0.0079 | 0.0215 |
| Osmolarity >308 mOsm/L | 0.0070 | 0.0000 |
| Schirmer test <3 mm/5 min | 0.0037 | 0.0343 |
| NIBUT <3 seconds | 0.0036 | 0.0395 |
| OSS discordant with symptoms > signs (score > +0.5) | 0.0035 | 0.0315 |
| Age >70 years | 0.0034 | 0.0000 |
| Moderate fluorescein corneal staining (Oxford ≥3) | 0.0032 | 0.0194 |
| NIBUT discordant with symptoms > signs (score > +0.5) | 0.0032 | 0.0062 |
| Blink rate <17 blinks/minute | 0.0030 | 0.0000 |
| NIBUT <5 seconds | 0.0026 | 0.0000 |
| Schirmer discordant with symptoms > signs (score > +0.5) | 0.0026 | 0.0459 |
| Cloudy with debris MG expression (by clinician) | 0.0026 | 0.0130 |
| Blink rate <12 blinks/minute | 0.0024 | 0.0000 |
| Moderate lissamine green staining (Oxford ≥3) | 0.0002 | 0.0081 |
| TMH <0.1 mm | 0.0000 | 0.0593 |
| TMH <0.2 mm | 0.0000 | 0.0650 |

OSDI: Ocular Surface Index score, OSS: Ocular surface staining, MG: Meibomian gland, NIBUT: Non-invasive tear break-uptime, TMH: Tear meniscus height
